# DNA Damage and Repair in Plants under Ultraviolet and Ionizing Radiations

**DOI:** 10.1155/2015/250158

**Published:** 2015-02-02

**Authors:** Sarvajeet S. Gill, Naser A. Anjum, Ritu Gill, Manoranjan Jha, Narendra Tuteja

**Affiliations:** ^1^Stress Physiology and Molecular Biology Lab, Centre for Biotechnology, MD University, Rohtak, Haryana 124 001, India; ^2^Centre for Environmental and Marine Studies (CESAM), Department of Chemistry, University of Aveiro, 3810-193 Aveiro, Portugal; ^3^Plant Molecular Biology Group, International Center for Genetic Engineering and Biotechnology (ICGEB), Aruna Asaf Ali Marg, New Delhi 110067, India

## Abstract

Being sessile, plants are continuously exposed to DNA-damaging agents present in the environment such as ultraviolet (UV) and ionizing radiations (IR). Sunlight acts as an energy source for photosynthetic plants; hence, avoidance of UV radiations (namely, UV-A, 315–400 nm; UV-B, 280–315 nm; and UV-C, <280 nm) is unpreventable. DNA in particular strongly absorbs UV-B; therefore, it is the most important target for UV-B induced damage. On the other hand, IR causes water radiolysis, which generates highly reactive hydroxyl radicals (OH^•^) and causes radiogenic damage to important cellular components. However, to maintain genomic integrity under UV/IR exposure, plants make use of several DNA repair mechanisms. In the light of recent breakthrough, the current minireview (a) introduces UV/IR and overviews UV/IR-mediated DNA damage products and (b) critically discusses the biochemistry and genetics of major pathways responsible for the repair of UV/IR-accrued DNA damage. The outcome of the discussion may be helpful in devising future research in the current context.

## 1. Introduction

Having sessile nature, plants have to cope with constant exposure of environmental stressors which includes UV-B, ozone, desiccation, rehydration, salinity, low and high temperature, and air and soil pollutants including metals-metalloids. Several chemical mutagens and crosslinking agents (e.g., mitomycin C, cisplatin), alkylating agents, aromatic compounds, ionizing radiations, and fungal and bacterial toxins are included as the other important environmental DNA-damaging agents [1]. Apart from severely impacting plant structural, enzymatic and nonenzymatic components, the aforesaid stressors may also negatively threaten plant genomes. Despite the very stable nature of plant genome, nuclear DNA is an inherently unstable molecule and can be damaged spontaneously, metabolically, or by aforesaid stress factors. The overproduction of reactive oxygen species (ROS) as byproducts of normal cellular metabolism or as a result of abiotic stress conditions leads to DNA damage in the cell [2, 3]. In addition to producing both single-strand DNA breaks (SSBs) and double-strand DNA breaks (DSBs), intrinsic DNA damage may include the loss of a base to form an abasic site, chemical modification of a base to form a miscoding or noncoding lesion, and sugar-phosphate backbone breakage [4, 5]. The accumulation of such damages (unrecognized and unrepaired DNA damage) may cause lethal mutations which in turn can reduce plant genome stability, growth, and productivity and also threaten the organism's immediate survival [2, 3, 5–7]. Therefore, effective detection of DNA damage, removal of damaged nucleotides, replacement with undamaged nucleotides* via* DNA synthesis, and repair of DNA damage are essential to eliminate the chance of permanent genetic alterations and hence to ensure the stability of the plant genome [2, 7, 8].

## 2. Ultraviolet (UV), Ionizing Radiations (IR), and Cellular DNA

Plants, because of their sessile nature, are especially susceptible to damage caused by environmental factors. Though sunlight is obligatory for photosynthesis and survival of plants, it also represents one of the major threats to their genomic integrity. Sunlight contains energy rich UV-A (320–400 nm), UV-B (290 to 320 nm), and UV-C (280 to 100 nm) light. UV-C is filtered out in the atmosphere and UV-B and UV-A can reach earth's surface effectively [3]. Among UV radiation types, UV-A radiation has been shown to have less DNA damaging effect because it cannot be absorbed by native DNA, whereas UV-A and visible light energy (up to 670–700 nm) can damage DNA* via* indirect photosensitizing reactions-mediated ROS generation especially singlet oxygen (^1^O_2_) [9]. UV-C radiation does not show much harmful effects on the biota since it is quantitatively absorbed by oxygen and ozone in the Earth's atmosphere. In the past 50 years, the concentration of ozone has decreased by about 5% (hence significant depletion in stratospheric ozone), mainly due to the release of anthropogenic pollutants such as chlorofluorocarbons [10]. Consequently, a larger proportion of the UV-B spectrum reaches the Earth's surface with serious implications on all living organisms [11–17].

**Figure 1:**
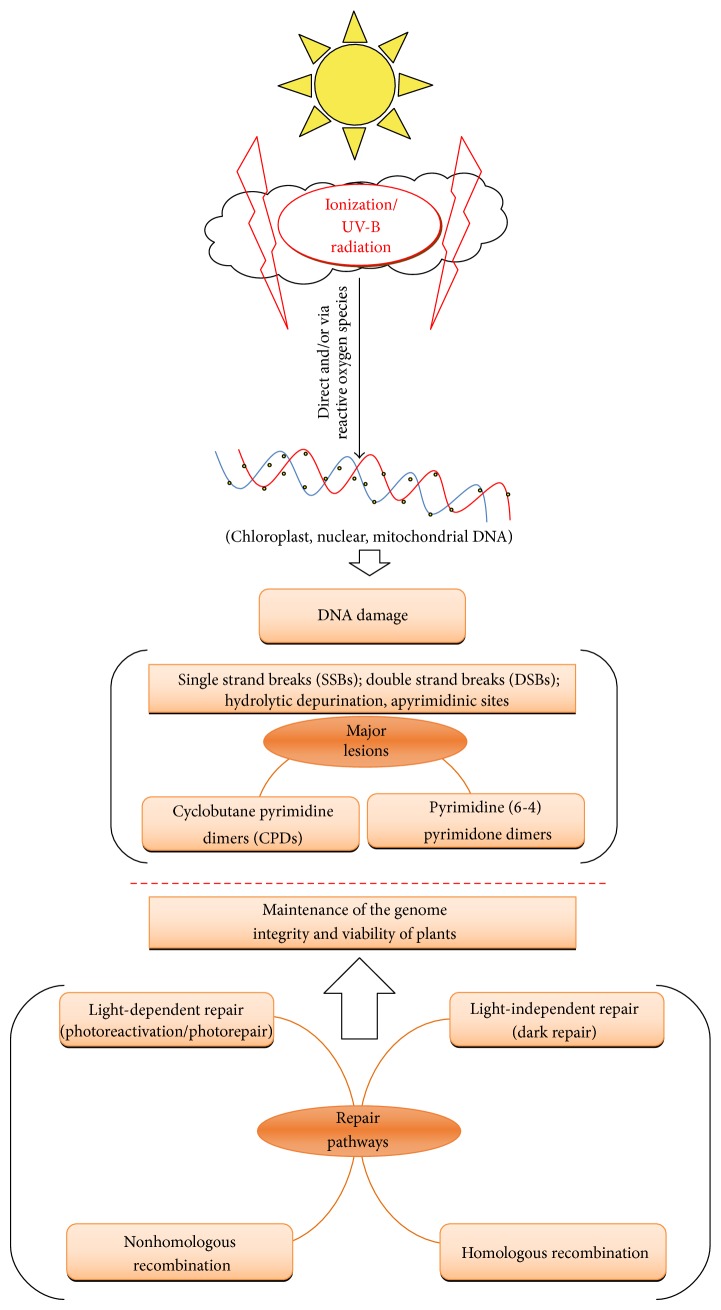
Mechanism of DNA damage and repair in plants.

UV-B radiation reaching the earth's surface is highly variable and influenced by many factors including stratospheric ozone layer and geographical area. Though most of the extraterrestrial UV-B is absorbed by the stratospheric ozone, remaining UV-B can produce adverse effects on diverse habitats [18]. Anthropogenically released chlorine and fluorine-containing compounds (e.g., CFCs) mediate significant depletion of stratospheric ozone which is basically responsible for increased UV-B radiation in the past decades in particular in the Antarctic zone [11, 12, 16, 17]. Although UV-B radiation has less than 1% of total solar energy, it is a highly active component of the solar radiation that brings chemical modification in DNA [19, 20]. UV radiation is one of the most damaging agents for DNA and other biomolecules such as proteins and lipids [21, 22]. Moreover, cellular DNA has been considered an obvious key target for UV induced genetic damage in a variety of organisms including plants [1, 23]. Mechanism of DNA damage and repair in response to UV/IR radiation has been shown in Figure 1.

On the perspective of IR mediated DNA damage in plants, it causes water radiolysis, which generates highly reactive OH^•^ radicals. OH^•^ radicals are the most reactive among all ROS and known to react with all biological molecules like DNA, proteins, lipids, and almost any constituent of cells. In the absence of any enzymatic mechanism for the removal of OH^•^, excess OH^•^ accumulation ultimately leads to cell death. DNA is the most preferred biomolecule being attacked by both the OH^•^ and the UV radiations (i.e., there is both direct and indirect effect) [24]. A number of alterations like SSBs and DSBs may take place in the IR and free radicals targeted DNA [24]. Plants may recruit a wide variety of strategies to either reverse, excise, or tolerate the presence of DNA damage products [5]. In this perspective, though mechanisms underlying UV and ionizing radiations-mediated DNA damage and repair have been thoroughly described in bacteria, yeast, and mammalian systems, information on these processes in plants is scanty and unsubstantiated. However, the DNA damage under UV radiation and DNA repair associated enzymes have been shown to greatly modulate the mutation rate, chromosome aberration frequencies, and viability in seeds and seedlings [25].

The following sections and subsections present an overview on UV/ionizing radiation-mediated DNA damage products and critically discuss potential, biochemical, and genetic mechanisms underlying UV/ionizing radiation accrued DNA damage and repair pathways.

## 3. UV-B Radiation Accrued DNA Damage

UV-B radiation can penetrate and damage plant genome by inducing oxidative damage (pyrimidine hydrates) and cross-links (both DNA protein and DNA-DNA) that are responsible for retarding the growth and development of plants [1, 6, 26, 27]. UV-B radiation damages nuclear, chloroplast, and mitochondrial DNA by inducing various DNA lesions including the generation of cyclobutane pyrimidine dimers (CPDs) (as the primary UV-B-induced DNA lesions accounting approximately 75% of UV-B-mediated total DNA damage) and other photoproducts, pyrimidine (6-4) pyrimidone dimers as the major lesions, while the minor includes oxidized or hydrated bases, single-strand breaks, and others [28, 29].

On the perspective of the production of different types of structural distortions within DNA due to CPDs and 6-4PPs, the induction of a slight bending on the DNA helix has been evidenced due to CPDs, whereas, 6-4PPs can produce much more bending and also unwinding on the DNA [1]. These DNA lesions together can act as the principal cause of UV-B-induced growth inhibition in plants. Additionally, these products can be lethal or mutagenic to organisms and can also impede transcriptional processes and result in error-prone replication [30–33]. Indirect generation of reactive oxygen species (ROS) due to solar UV light has also been evidenced in the nucleus [34]. Nevertheless, ROS-accrued base and nucleotide modifications, especially in sequences with high guanosine content, and also strand breaks have been widely reported and reviewed [6, 30, 35, 36].

### 3.1. Cyclobutane Pyrimidine Dimer

Cyclobutane pyrimidine dimer (CPD) is a major type of DNA damage induced by UV-B radiation. CPDs constitute the majority of these lesions (approximately 75%). Herein, any of the diastereoisomers such as cis/trans (relative position of pyrimidine rings) and syn-anti (relative orientation of C5–C6 bonds) can be formed. Moreover, though cis-syn forms of CPDs are of common occurrence, trans-syn forms are present exclusively within single-stranded DNA [1, 37]. No or very low amounts of CPDs have been evidenced due to low UV-B rates (<1 *μ*mol m^−2^ s^−1^) which though below the limit of detection, stimulate protective and photomorphogenetic responses [38, 39], eventually affecting the plant's resistance to UV-B stress [15, 28, 38].

### 3.2. Pyrimidine (6-4) Pyrimidone Dimers

Pyrimidine (6-4) pyrimidone dimers (6-4PPs dimers) are other major lesions caused by UV-B radiation. However, the occurrence of 6-4PPs dimers is much less frequent than CPDs [6, 40]. The UV radiation wavelength and sequence dependent formation of the 6-4PPs at adjacent TT, TC, and CC nucleotides has also been evidenced [1]. To this end, in addition to being impacted by UV-B, 6-4PP dimers are photoisomerized by UV-A light resulting in the Dewar isomer [37, 40].

### 3.3. Ionizing Radiation and DNA Damage

A number of alterations may take place in the IR and free radicals targeted DNA like single-strand breaks and double-strand breaks, and hydrolytic depurination, apyrimidinic sites, and oxidative damage to the bases and the phosphodiester backbone of DNA are common [24, 41]. Gamma irradiation with different dose rates induces different DNA damage responses in Petunia × hybrida cells [42]. Double-strand breaks (DSBs) are a strikingly genotoxic form of DNA damage; hence, DSBs present a unique challenge to the cell. The failure to repair a break in DNA has been widely evidenced to cause loss of a chromosome arm where misrepair can produce translocations, deletions, and other chromosomal abnormalities [43, 44]. In addition, unrepaired breaks in dividing cells can initiate cell cycle checkpoints, halting division, and, consequently, slowing the growth of the organism [45]. However, the production of aneuploid daughter cells (that are often not viable) has been reported as nondividing cells with DSBs [46, 47]. The developmental regulation of DSB repair mechanisms in plants has also been evidenced [48]. The occurrence of multiple pathways for the repair of DSBs has been reported in plants [49]. Some of the major pathways for DSB-repair in plants will be overviewed in the following sections.

## 4. DNA Damage-Repair Pathways

The repair of DNA damage is essential for the survival of organisms while, if repair does not take place, genomic integrity will not be maintained [50]. To this end, coordination between DNA replication and repair has been considered essential for the maintenance of the genome [51]. The UV radiation induced DNA damage and repair has been well studied but concrete information on the underlying mechanisms in plant system is still lacking [51].

Information pertaining to the dynamics of DNA damage accumulation and molecular mechanisms that regulate recovery from radiation injury as a function of dose rate is unsubstantiated and poorly explored [24, 42]. Nevertheless, the information to date available in the current context indicates that a mechanism of DNA damage repair in plants is very complex which actually involves multiple loci. Additionally, involvement of mechanisms—either similar or identical to those regulating the integrity of foreign sequences in the plant genomes—has been reported [24]. In addition, plant response to IR may also involve the activation of cell cycle check-points which subsequently may lead to the DNA repair response [52]. The studies on *γ*-rays on* Populus nigra* var. italica revealed that the modulation of repair mechanisms is essential for the adaptive response to IR [53]. Chronic or acute irradiation exposed* Arabidopsis thaliana* plants exhibited differential regulation of gene expression of DNA repair machinery [54]. It is to be underlined here that the DNA damage contributed by either UV and/or IR can be corrected employing a number of pathways such as direct repair, photorepair (photoreactivation), base excision repair (BER), nucleotide excision repair (NER), or mismatch repair (MMR) in plants where abovementioned lesion-specific repair pathways can reverse UV/IR-induced DNA damage to preserve the genomic integrity [55–58]. The significance of the replicative arrest avoidance has also been evident in plants as a potential mechanism for UV photoproducts-tolerance. Moreover, implication of genes isolated from the model plant* A. thaliana* in nucleotide excision repair or tolerance of UV-induced DNA damage was a possible result of phenotypic characterization of plant mutants, functional complementation studies, and cDNA analysis [57].

Major mechanisms underlying coping strategies for UV-B/IR-induced DNA damage are being overviewed hereunder.

### 4.1. Light-Dependent Repair (Photoreactivation/Photorepair)

The photorepair (photoreactivation) is the major pathway in plants for repairing UV-B induced low frequencies of DNA damage (especially of UV-B induced CPDs) where the photolyase mediates the major processes by absorbing blue/UV-A (320–400 nm) light and uses the energy to monomerize dimers [1, 59–62]. There exist similar reaction mechanisms underlying both CPD photolyases and 6-4 photolyases-mediated repair of the respective pyrimidine dimers [40, 63]. The occurrence of the reduced pterin MTHF in plant-CPD photolyase activity as the second chromophore has been evidenced [64]. Photolyases bind specifically to DNA lesions and efficiently and quickly remove the bulk of UV-induced CPDs and (6-4)-photoproducts directly by absorbing light in the 300–600 nm range [27, 65, 66]. A number of cofactors including the quality, timing, and quantity of photoreactivating light and damage levels largely modulate this repair [60, 65, 67]. Different genotypes exhibiting sensitivity to UV-B were reported to exhibit differential ability to repair UV-B-mediated DNA damage types (such as CPDs), where UV-B = sensitive cultivars were less able to repair CPDs through photoreactivation than UV-B resistant cultivars [68].

To date, photoreactivation and photolyases have been extensively reported in several plant species [65–77]. However, credible work has been performed on* Oryza sativa* cultivars, where photolyase has been evidenced as a major factor modulating* O. sativa* cultivar capability to repair DNA damage types [68, 76–79]. Earlier also, the sensitivity of* O. sativa* to UV-B radiation was reported to vary among cultivars [78]. Based on the exhibition of* O. sativa* cultivars potential for the level of CPD photolyase activity,* O. sativa* cultivars were clearly classified into three groups, which in turn depended on amino acid residues at positions 126 (UV-B resistant* O. sativa*, glutamine; UV-B sensitive and UV-B hypersensitive* O. sativa*, arginine) and 296 (UV-B resistant and UV-B sensitive* O. sativa*, glutamine; UV-B hypersensitive, histidine) [68]. It was also postulated that increasing the activity of the photolyase enzyme in* O. sativa* may increase their resistance to UV-B radiation [68, 77, 79]. In some recent reports, UV-B sensitive* O. sativa* cultivars were evidenced to exhibit less capability to repair CPDs through photoreactivation than UV-B resistant cultivars, where the authors considered an alteration of CPD photolyase activity resulting from spontaneously occurring mutations in the CPD photolyase gene [68, 76, 77, 79]. Transgenic* O. sativa* plants have also been successfully generated bearing the CPD photolyase gene from UV-B resistant* O. sativa* cultivars [68, 80]. Overexpression of CPD photolyase in* O. sativa* results in higher CPD photolyase activity which resulted in significant more resistance to UV-B induced growth damage than wild-type plants. In contrast, plants with the gene transferred in the antisense orientation had significantly lower CPD photolyase activity and showed less resistance to UV-B radiation. Recently, Teranishi et al. [68] developed CPD photolyase overexpressing transgenic* O. sativa* plants with higher CPD photolyase activity using UV-B sensitive* O*.* sativa* Norin 1 (japonica) and UV-B hypersensitive* O. sativa* Surjamkhi (indica) as parental line (PL) plants. These results emphasized that CPD photolyase is a crucial factor for determining UV-B sensitivity in* O. sativa* [68, 80]. In addition to the studies on* O. sativa*, the overexpression of CPD photolyase in* A. thaliana* also resulted in a moderate increase in biomass production under conditions of elevated UV-B radiation [17]. Moreover, an ecotype-specific genetic variability in the UV-B response in* A. thaliana* has also been evidenced [81].

### 4.2. Light-Independent Repair (Dark Repair)

The dark repair includes nucleotide excision repair (NER), base excision repair (BER), mismatch repair (MMR), and other DNA repair pathways. Excision repair (NER and BER) is very important for maintaining genome stability and essential for the survival of organisms. The potential mechanisms underlying dark repair reactions have been discussed earlier extensively [40, 82, 83]. The occurrence of light-independent repair pathway has been evidenced in a number of plant species including carrot protoplasts [84, 85],* A. thaliana* [86], alfalfa seedlings [59, 65], soybean chloroplasts and leaves [60, 87],* O. sativa* cultivars [61], and* Triticum aestivum* leaf tissues [88] (reviewed by Britt et al. [71] & Tuteja et al. [6]). Despite the availability of biochemical and genetic data in favor of some sort of NER in plants, limited information is available concerning the molecular characterization of plant gene products actively involved in dark repair [62]. Compared to the light-dependent repair (photoreactivation/photorepair), more general and flexible feature has been evidenced by the light-independent repair (dark repair) where the recognition and targeting of the damaged strand and subsequent removal of a 24–32 base oligonucleotide containing the damaged product and filling the gap through DNA synthesis and ligation of the nicks have been reported [89, 90]. In context with NER, this system has been considered the most versatile system for dealing with the DNA damage [91]. Since NER recognizes conformational changes in the DNA duplex rather than a specific type of DNA damage, this system can also repair different types of damage [25]. Moreover, NER comprises the two subpathways, namely, global genomic repair (GGR) and transcription-coupled repair (TCR), where GGR repairs the DNA damage over the entire genome and TCR is selective for the transcribed DNA strand in expressed genes [51]. The wide class of helix-distorting lesions such as CPDs and (6-4) photoproducts are repaired by NER [6, 27]. The sequential involvement of NER-mediated recognition of DNA damage, incision on damaged strand, excision of damage containing oligonucleotides, DNA synthesis, and ligation has been reported [51]. The conserved nature of the NER repair pathway has been proved by revealing most of the genes involved in NER in* A*.* thaliana* [92].

BER also comprises the two subpathways, namely, the short-patch BER and long-patch BER, where the former is DNA polymerase (beta-dependent) and the latter is DNA polymerase (delta/epsilon-dependent). BER repairs the oxidized or hydrated bases and SSB. Herein, DNA glycosylases initiate this process by releasing the damaged base, with cleavage of the sugar phosphate chain, excision of a basic residue or of a basic residue containing oligonucleotides, and DNA synthesis and ligation [6, 27]. Short-patch BER is initiated by removal of the damaged base by a DNA glycosylase enzyme that is specific for the particular base adduct [93, 94], whereas in the long-patch BER, the repair is a result of nick translation reaction accompanied by strand displacement in the 5′–3′ direction, thereby generating a flap type of structure [95, 96]. The homologues of components involved in BER in* O. sativa* and* A*.* thaliana* have also been reported [51, 97–99].

Insertions or deletions of nucleotides (potential frame shift mutations) may be more frequent, where nucleotide-repeat sequences can give rise to slip-mispairing [100]. Additionally, the mismatched bases also arise during recombination. However, the mismatch repair (MMR) systems have been evolved to correct a large portion of these errors, further reducing the error rate 10^−9^ to 10^−10^ [101]. The promotion of genomic stability by highly conserved MMR systems* via* the correction of DNA replication errors, antagonizing homeologous recombination and responding to various DNA lesions, has been widely evidenced [101]. MMR mechanism is highly conserved between species where it removes majority (99.9%) of the errors remaining after polymerase proofreading to reduce the error rate to one misincorporated base per 10^9^-10^10^ nucleotides in the nascent DNA chain. Moreover, MMR may also recognize mismatches at sites of recombination between DNA sequences; thereby it can reduce the rate of occurrence of recombination events [101, 102]. Encoding a suite of MMR protein orthologs, including MSH2, the constant component of various specialized eukaryotic mismatch recognition heterodimers has been reported in* A*.* thaliana* and other plants [101].

### 4.3. Double-Strand Breaks Repair

Double-strand breaks (DSBs) have to be eliminated before genomes can be replicated; hence, genomic DSBs have been regarded as key intermediates in recombination reactions of living organisms. Nevertheless, the efficient repair of genomic DNA-DSBs is important for the survival of all organisms [103, 104]. Though the studies on DSBs repair in animals and yeast have been credibly performed, this aspect in plants lags behind [44]. In recent years, basic mechanisms of DSB repair in somatic plant cells have been elucidated. In addition, homologous recombination (HR) and the nonhomologous system (NHR) are the two general classes of recombination to perform DSBs-repair. The availability of complete sequences of* A*.* thaliana* and* O. sativa* has intensified efforts to characterize endogenous regulatory components of HR and nonhomologous end joining (NHEJ) [92]. HR and NHR-assisted repair of double-strand DNA breaks has been reported to control the viability under irradiation [104, 105]. Both HR and NHR/NHEJ pathways are responsible for balancing genome stability against the generation of genetic diversity. Additionally, the essential roles of HR and NHEJ have been considered vital for genetic engineering [44]. Moreover, there are credible reports on the (a) isolation of radiation-sensitive plant mutants [27, 40, 106, 107] and (b) successful cloning of many plant genes from different repair pathways involved in repair of radiation-induced damage [108–111] but to date, the efficiency of NER and DSB repair and the stability/modification of these systems under chronic irradiation conditions have not been reported [25]. Some of the specific features and overall significance of these two DSB-repair pathways (i.e., HR and NHEJ) are highlighted hereunder.

### 4.4. Homologous Recombination

HR has been considered as the major pathway for maintaining the genome integrity and viability of plants where it performs mainly the error-free DSB-repair. HR uses a homologous chromosome or chromatid as a template to recover information [43]. Moreover, in HR, the double-stranded gap generated by a frayed DSB is filled by copying, or in some cases splicing, homologous sequences from elsewhere in the genome [44]. A credible work in yeast has led to the categorization of models pertaining to HR into three models: DNA double-strand break repair (DSBR) model, synthesis-dependent strand annealing (SDSA), and single-strand annealing (SSA) [40, 112, 113]. In brief, both DSBR and SDSA are initiated by a 3′ resection forming a long single-stranded DNA tail that invades a homologous duplex and primes DNA synthesis but in SDSA, the newly synthesized DNA then reanneals with the other side of the DSB, repairing the break and avoiding the formation of the joint molecule [40]. SSA may occur between tandemly repeated sequences and anneal homologous regions exposed during resection. A number of plant species have been evidenced to possess DSBR, SDSA, and SSA HR pathways exhibiting much of the common molecular machineries, where recombination frequencies were reported to greatly vary from the origin of the donor and recipient sequences [40, 104, 114]. Very rare (around 1 in 10,000 repair events) occurrence of HR-mediated DSB repair has been reported in plant somatic cells* via* allelic sequences [115].

On the perspective of molecular mechanism of HR, a number of researchers including [40, 114, 116] reported or reviewed sharing of similarities in meiotic and somatic recombination pathways at the molecular level. Central to the process of homologous recombination are the RAD52 epistasis group genes (*RAD50, RAD51, RAD52, RAD54, RDH54/TID1, RAD55, RAD57, RAD59, MRE11*, and* XRS2*), most of which were identified by their requirement for the repair of IR-induced DNA damage in yeast. The contribution of mutations in these genes was evidenced to cause defects in meiotic and/or mitotic recombination and thus has provided evidence for a link between DSB repair (DSBR) and HR [117]. Among these genes, owing to severe meiotic defects,* A*.* thaliana* HR null mutants such as* AtRAD51, AtRAD50,* and* AtMRE11* were reported to be completely sterile [108, 118]. In addition, meiocytes of these mutant lines were evidenced to show extensive chromosome fragmentation leading ultimately to nonviable gametes because of the incapability of these mutants to align homologous chromosomes (synapsis) during the early stages of meiosis [108, 118].* Rad54* has been isolated and characterized from* A. thaliana*, where its significance in HR was advocated [119]. A weaker expression of* Rad54* has been evidenced earlier in irradiated* A. thaliana* when compared to nonirradiated plants. A plant homolog of Nijmegen breakage syndrome-1 gene from* Arabidopsis* (*AtNbs1*) and* O. sativa* (*OsNbs1*) has been identified and their involvement in DNA/DSB repair was evidenced [120–122].

### 4.5. Nonhomologous Recombination

The nonhomologous recombination system or nonhomologous end-joining (NHR/NHEJ) system, also termed as “illegitimate recombination,” does not require homologous sequences; rather, it acts to rejoin the two end breaks and often results in deletions or mistakes and thus mutations. Although NHR is obviously error prone and degraded or even inappropriate ends may be rejoined, this repair system appears to be crucial in radio-induced DSB repair in plants [40, 43, 44]. Even though HR has been evidenced less efficient in DSB repair in plants, the information on NHR (or NHEJ) in plants is lacking [123]; thus, it is important to have insight into NHR pathway since genomic alterations in meristematic cells can be transferred to the offspring [104]. In somatic tissue, NHEJ has been evidenced as a major pathway for DSB repair. Since NHEJ-mediated rejoining of the broken ends is associated with deletions of various sizes and also with insertions of sequences (filler DNA) (that are often copied from sites close to the DSB) and sequence similarities are not required in NHEJ for the incorporation of filler DNA into the break; NHEJ does not preserve genetic information and genomic integrity at the break site [124]. NHEJ may also differ among species where an inverse correlation of deletion size to genome size has been postulated; thus, NHEJ significance in the genome size evolution has been advocated [125]. Details about the molecular-genetics of NHEJ in eukaryotes can be found elsewhere [126].

## 5. Conclusion and Future Perspectives

Plant nuclear DNA is an inherently unstable molecule and can be damaged spontaneously, metabolically, or by a number of stress factors. Though sunlight is obligatory for photosynthesis and survival of plants, it also represents one of the major threats to their genomic integrity. Sunlight contains energy rich UV-A (320–400 nm), UV-B (290–320 nm), and UV-C (280–100 nm) light. While UV-C is filtered out in the atmosphere, UV-B and UV-A can reach earth's surface. On the other hand, IR causes water radiolysis, which generates highly reactive hydroxyl radicals. DNA is the object of an attack by both UV and IR radiations leading to a number of alterations including SSBs and DSBs. The accumulation of such damages and unrecognized and unrepaired DNA damage may cause fatal mutations which in turn can reduce plant genome stability, growth, and productivity and also threaten the organism's immediate survival. Plants employ various strategies to either reverse, excise, or tolerate the presence of DNA damage products. The literature reviewed here reflected the paucity of information on the basic mechanisms underlying UV or IR mediated DNA damage and repair compared to bacteria, yeast, and mammalian systems. Both HR and NHR pathways-mediated facilitation of the programmed repair of DSBs have been evidenced. Thus, plants have become an ideal system for the identification of genes which are not accessible to classical genetic analysis in other systems (including mammalian model systems). Identification and characterization of UV/IR-sensitive mutants in different plant species are expected to achieve more insights into genetic recombination/manipulation in plants.

## References

[1] Tuteja N., Ahmad P., Panda B. B., Tuteja R. (2009). Genotoxic stress in plants: shedding light on DNA damage, repair and DNA repair helicases. *Mutation Research: Reviews in Mutation Research*.

[2] Gill S. S., Tuteja N. (2010). Reactive oxygen species and antioxidant machinery in abiotic stress tolerance in crop plants. *Plant Physiology and Biochemistry*.

[3] Biedermann S., Mooney S., Hellmann H., Chen C. (2011). Recognition and repair pathways of damaged DNA in higher plants. *Selected Topics in DNA Repair*.

[4] Vonarx E. J. (2000). *The repair and tolerance of DNA damage in higher plants [Ph.D. thesis]*.

[5] Singh S. K., Roy S., Choudhury S. R., Sengupta D. N. (2010). DNA repair and recombination in higher plants: insights from comparative genomics of arabidopsis and rice. *BMC Genomics*.

[6] Tuteja N., Singh M. B., Misra M. K., Bhalla P. L., Tuteja R. (2001). Molecular mechanisms of DNA damage and repair: progress in plants. *Critical Reviews in Biochemistry and Molecular Biology*.

[7] Waterworth W. M., Drury G. E., Bray C. M., West C. E. (2011). Repairing breaks in the plant genome: the importance of keeping it together. *New Phytologist*.

[8] Roy S., Singh S. K., Choudhury S. R., Sengupta D. N. (2009). An insight into the biological functions of family X-DNA polymerase in DNA replication and repair of plant genome. *Plant Signaling and Behavior*.

[9] Alscher R. G., Donahue J. L., Cramer C. L. (1997). Reactive oxygen species and antioxidants: relationships in green cells. *Physiologia Plantarum*.

[10] Pyle J. A., Lumbsden P. J. (1996). Global ozone depletion: observation and theory. *Plants and UV-B. Responses to Environmental Change*.

[11] Anderson J. G., Toohey D. W., Brune W. H. (1991). Free radicals within the Antarctic vortex: the role of CFCs in Antarctic ozone loss. *Science*.

[12] McKenzie R., Connor B., Bodeker G. (1999). Increased summertime UV radiation in New Zealand in response to ozone loss. *Science*.

[13] Xiong F. S., Day T. A. (2001). Effect of solar ultraviolet-B radiation during springtime ozone depletion on photosynthesis and biomass production of Antarctic vascular plants. *Plant Physiology*.

[14] Cannon G. C., Hedrick L. A., Heinhorst S. (1995). Repair mechanisms of UV-induced DNA damage in soybean chloroplasts. *Plant Molecular Biology*.

[15] Frohnmeyer H., Staiger D. (2003). Ultraviolet-B radiation-mediated responses in plants. Balancing damage and protection. *Plant Physiology*.

[16] Solomon S., Qin D., Manning M. (2007). *IPCC 2007: Climate Change 2007: The Physical Science Basis. Contribution of Working Group I to the Fourth Assessment Report of the Intergovernmental Panel on Climate Change*.

[17] Kaiser G., Kleiner O., Beisswenger C., Batschauer A. (2009). Increased DNA repair in *Arabidopsis* plants overexpressing CPD photolyase. *Planta*.

[18] McKenzie R. L., Björn L. O., Bais A., Ilyasd M. (2003). Changes in biologically active ultraviolet radiation reaching the Earth's surface. *Photochemical and Photobiological Sciences*.

[19] Sinha R. P., Häder D.-P. (2002). UV-induced DNA damage and repair: a review. *Photochemical & Photobiological Sciences*.

[20] Rastogi R. P., Kumar A., Tyagi M. B., Sinha R. P. (2010). Molecular mechanisms of ultraviolet radiation-induced DNA damage and repair. *Journal of Nucleic Acids*.

[21] Prinsze C., Dubbelman T. M. A. R., van Steveninck J. (1990). Protein damage, induced by small amounts of photodynamically generated singlet oxygen or hydroxyl radicals. *Biochimica et Biophysica Acta*.

[22] Smirnoff N., Smirnoff N. (1995). Antioxidant systems and plant response to the environment. *Environment and Plant Metabolism, Flexibility and Acclimation*.

[23] Quaite F. E., Sutherland B. M., Sutherland J. C. (1992). Quantitation of pyrimidine dimers in DNA from UVB-irradiated alfalfa (*Medicago sativa* L.) seedlings. *Applied and Theoretical Electrophoresis*.

[24] Esnault M.-A., Legue F., Chenal C. (2010). Molecular mechanisms of ultraviolet radiation-induced DNA damage and repair. *Environmental and Experimental Botany*.

[25] Boubriak I. I., Grodzinsky D. M., Polischuk V. P. (2008). Adaptation and impairment of DNA repair function in pollen of *Betula verrucosa* and seeds of *Oenothera biennis* from differently radionuclide- contaminated sites of Chernobyl. *Annals of Botany*.

[26] Stapleton A. E. (1992). Ultraviolet radiation and plants: burning questions. *The Plant Cell*.

[27] Britt A. B. (1999). Molecular genetics of DNA repair in higher plants. *Trends in Plant Science*.

[28] Ballaré C. L., Rousseaux M. C., Searles P. S. (2001). Impacts of solar ultraviolet-B radiation on terrestrial ecosystems of Tierra del Fuego (southern Argentina)—an overview of recent progress. *Journal of Photochemistry and Photobiology B: Biology*.

[29] Takahashi M., Teranishi M., Ishida H. (2011). Cyclobutane pyrimidine dimer (CPD) photolyase repairs ultraviolet-B-induced CPDs in rice chloroplast and mitochondrial DNA. *The Plant Journal*.

[30] Demple B., Harrison L. (1994). Repair of oxidative damage to DNA: enzymology and biology. *Annual Review of Biochemistry*.

[31] Hoeijmakers J. H. (2001). Genome maintenance mechanisms for preventing cancer. *Nature*.

[32] Sancar A., Lindsey-Boltz L. A., Ünsal-Kaçmaz K., Linn S. (2004). Molecular mechanisms of mammalian DNA repair and the DNA damage checkpoints. *Annual Review of Biochemistry*.

[33] Friedberg E. C., Walker G. C., Siede W., Wood R. D., Schultz R. A., Ellenberger T. (2006). *DNA Repair and Mutagenesis*.

[34] Iovine B., Nino M., Irace C., Bevilacqua M. A., Monfrecola G. (2009). Ultraviolet B and A irradiation induces fibromodulin expression in human fibroblasts *in vitro*. *Biochimie*.

[35] Wiseman H., Halliwell B. (1996). Damage to DNA by reactive oxygen and nitrogen species: role in inflammatory disease and progression to cancer. *Biochemical Journal*.

[36] Tuteja N., Tuteja R. (2001). Unraveling DNA repair in human: molecular mechanisms and consequences of repair defect. *Critical Reviews in Biochemistry and Molecular Biology*.

[37] Mouret S., Baudouin C., Charveron M., Favier A., Cadet J., Douki T. (2006). Cyclobutane pyrimidine dimers are predominant DNA lesions in whole human skin exposed to UVA radiation. *Proceedings of the National Academy of Sciences of the United States of America*.

[38] Kim B. C., Tennessen D. J., Last R. L. (1998). UV-B-induced photomorphogenesis in *Arabidopsis thaliana*. *The Plant Journal*.

[39] Frohnmeyer H., Loyall L., Blatt M. R., Grabov A. (1999). Millisecond UV-B irradiation evokes prolonged elevation of cytosolic-free Ca^2+^ and stimulates gene expression in transgenic parsley cell cultures. *Plant Journal*.

[40] Bray C. M., West C. E. (2005). DNA repair mechanisms in plants: crucial sensors and effectors for the maintenance of genome integrity. *New Phytologist*.

[41] Slupphaug G., Kavli B., Krokan H. E. (2003). The interacting pathways for prevention and repair of oxidative DNA damage. *Mutation Research*.

[42] Donà M., Ventura L., Macovei A. (2013). Gamma irradiation with different dose rates induces different DNA damage responses in *Petunia x hybrida* cells. *Journal of Plant Physiology*.

[43] Friesner J., Britt A. B. (2003). *Ku80*- and *DNA ligase IV*-deficient plants are sensitive to ionizing radiation and defective in T-DNA integration. *Plant Journal*.

[44] Hefner E., Preuss S. B., Britt A. B. (2003). Arabidopsis mutants sensitive to gamma radiation include the homologue of the human repair gene ERCC1. *Journal of Experimental Botany*.

[45] Lydall D., Weinert T. (1995). Yeast checkpoint genes in DNA damage processing: implications for repair and arrest. *Science*.

[46] Belyakov O. V., Prise K. M., Trott K. R., Michael B. D. (1999). Delayed lethality, apoptosis and micronucleus formation in human fibroblasts irradiated with X-rays or *α*-particles. *International Journal of Radiation Biology*.

[47] Morgan W. F., Day J. P., Kaplan M. I., McGhee E. M., Limoli C. L. (1996). Genomic instability induced by ionizing radiation. *Radiation Research*.

[48] Boyko A., Zemp F., Filkowski J., Kovalchuk I. (2006). Double-strand break repair in plants is developmentally regulated. *Plant Physiology*.

[49] Friedberg E. C., Walker G. C., Siede W. (1995). *DNA Repair and Mutagenesis*.

[50] Ries G., Heller W., Puchta H., Sandermann H., Seldlitz H. K., Hohn B. (2000). Elevated UV-B radiation reduces genome stability in plants. *Nature*.

[51] Kimura S., Tahira Y., Ishibashi T. (2004). DNA repair in higher plants; photoreactivation is the major DNA repair pathway in non-proliferating cells while excision repair (nucleotide excision repair and base excision repair) is active in proliferating cells. *Nucleic Acids Research*.

[52] Cools T., de Veylder L. (2009). DNA stress checkpoint control and plant development. *Current Opinion in Plant Biology*.

[53] Nishiguchi M., Nanjo T., Yoshida K. (2012). The effects of gamma irradiation on growth and expression of genes encoding DNA repair-related proteins in Lombardy poplar (*Populus nigra* var. italica). *Journal of Environmental Radioactivity*.

[54] Kovalchuk I., Molinier J., Yao Y., Arkhipov A., Kovalchuk O. (2007). Transcriptome analysis reveals fundamental differences in plant response to acute and chronic exposure to ionizing radiation. *Mutation Research*.

[55] Norbury C. J., Hickson I. D. (2001). Cellular responses to DNA damage. *Annual Review of Pharmacology and Toxicology*.

[56] Molinier J., Stamm M.-E., Hohn B. (2004). SNM-dependent recombinational repair of oxidatively induced DNA damage in *Arabidopsis thaliana*. *EMBO Reports*.

[57] Kunz B. A., Anderson H. J., Osmond M. J., Vonarx E. J. (2005). Components of nucleotide excision repair and DNA damage tolerance in *Arabidopsis thaliana*. *Environmental and Molecular Mutagenesis*.

[58] Zhang Y., Rohde L. H., Emami K. (2008). Suppressed expression of non-DSB repair genes inhibits gamma-radiation-induced cytogenetic repair and cell cycle arrest. *DNA Repair*.

[59] Quaite F. E., Takayanagi S., Ruffini J., Sutherland J. C., Sutherland B. M. (1994). DNA damage levels determine cyclobutyl pyrimidine dimer repair mechanisms in alfalfa seedlings. *Plant Cell*.

[60] Sutherland B. M., Takayanagi S., Sullivan J. H., Sutherland J. C. (1996). Plant responses to changing environmental stress: cyclobutyl pyrimidine dimer repair in soybean leaves. *Photochemistry and Photobiology*.

[61] Hidema J., Kumagai T., Sutherland J. C., Sutherland B. M. (1997). Ultraviolet B-sensitive rice cultivar deficient in cyclobutyl pyrimidine dimer repair. *Plant Physiology*.

[62] Gallego F., Fleck O., Li A., Wyrzykowska J., Tinland B. (2000). *AtRAD1*, a plant homologue of human and yeast nucleotide excision repair endonucleases, is involved in dark repair of UV damages and recombination. *Plant Journal*.

[63] Sancar A. (2003). Structure and function of DNA photolyase and cryptochrome blue-light photoreceptors. *Chemical Reviews*.

[64] Waterworth W. M., Jiang Q., West C. E., Nikaido M., Bray C. M. (2002). Characterization of *Arabidopsis* photolyase enzymes and analysis of their role in protection from ultraviolet-B radiation. *The Journal of Experimental Botany*.

[65] Pang Q., Hays J. B. (1991). UV-B-inducible and temperature-sensitive photoreactivation of cyclobutane pyrimidine dimers in *Arabidopsis thaliana*. *Plant Physiology*.

[66] Takeuchi Y., Murakami M., Nakajima N., Kondo N., Nikaido O., Takeuchi Y. (1996). The photorepair of photoisomerization of DNA lesions in etiolated cucumber cotyledons after irradiation by UV-B depends on wavelength. *Plant Cell Physiology*.

[67] Stapleton A. E., Thornber C. S., Walbot V. (1997). UV-B component of sunlight causes measurable damage in field-grown maize (*Zea mays* L.): developmental and cellular heterogeneity of damage and repair. *Plant, Cell & Environment*.

[68] Teranishi M., Taguchi T., Ono T., Hidema J. (2012). Augmentation of CPD photolyase activity in japonica and indica rice increases their UVB resistance but still leaves the difference in their sensitivities. *Photochemical and Photobiological Sciences*.

[69] Hidema J., Kumagai T. (1998). UVB-induced cyclobutyl pyrimidine dimer and photorepair with progress of growth and leaf age in rice. *Journal of Photochemistry and Photobiology B*.

[70] Kumagai T., Hidema J., Kang H.-S., Sato T. (2001). Effects of supplemental UV-B radiation on the growth and yield of two cultivars of Japanese lowland rice (*Oryza sativa* L.) under the field in a cool rice-growing region of Japan. *Agriculture, Ecosystems & Environment*.

[71] Britt A. B., Chen J.-J., Wykoff D., Mitchell D. (1993). A UV-sensitive mutant of Arabidopsis defective in the repair of pyrimidine-pyrimidinone(6–4) dimers. *Science*.

[72] Hidema J., Kumagai T., Sutherland B. M. (2000). UV radiation-sensitive Norin 1 rice contains defective cyclobutane pyrimidine dimer photolyase. *Plant Cell*.

[73] Dany A.-L., Douki T., Triantaphylides C., Cadet J. (2001). Repair of the main UV-induced thymine dimeric lesions within *Arabidopsis thaliana* DNA: evidence for the major involvement of photoreactivation pathways. *Journal of Photochemistry and Photobiology B: Biology*.

[74] Takahashi S., Nakajima N., Saji H., Kondo N. (2002). Diurnal change of cucumber CPD photolyase gene (*CsPHR*) expression and its physiological role in growth under UV-B irradiation. *Plant and Cell Physiology*.

[75] Tanaka A., Sakamoto A., Ishigaki Y. (2002). An ultraviolet-B-resistant mutant with enhanced DNA repair in arabidopsis. *Plant Physiology*.

[76] Teranishi M., Iwamatsu Y., Hidema J., Kumagai T. (2004). Ultraviolet-B sensitivities in Japanese lowland rice cultivars: cyclobutane pyrimidine dimer photolyase activity and gene mutation. *Plant and Cell Physiology*.

[77] Yamamoto A., Hirouchi T., Mori T. (2007). Biochemical and biological properties of DNA photolyases derived from utraviolet-sensitive rice cultivars. *Genes & Genetic Systems*.

[78] Kumagai T., Sato T. (1992). Inhibitory effects of increase in near-UV radiation on the growth of Japanese rice cultivars (*Oryza sativa* L.) in a phytotron and recovery by exposure to visible radiation. *Japan Journal of Breeding*.

[79] Hidema J., Teranishi M., Iwamatsu Y. (2005). Spontaneously occurring mutations in the cyclobutane pyrimidine dimer photolyase gene cause different sensitivities to ultraviolet-B in rice. *Plant Journal*.

[80] Hidema J., Taguchi T., Ono T., Teranishi M., Yamamoto K., Kumagai T. (2007). Increase in CPD photolyase activity functions effectively to prevent growth inhibition caused by UVB radiation. *Plant Journal*.

[81] Kalbina I., Strid Å. (2006). Supplementary ultraviolet-B irradiation reveals differences in stress responses between *Arabidopsis thaliana* ecotypes. *Plant, Cell and Environment*.

[82] Berg B. J. V., Sancar G. B. (1998). Evidence for dinucleotide flipping by DNA photolyase. *The Journal of Biological Chemistry*.

[83] Carell T., Burgdorf L. T., Kundu L. M., Cichon M. (2001). The mechanism of action of DNA photolyases. *Current Opinion in Chemical Biology*.

[84] Howland G. P. (1975). Dark repair of ultraviolet induced pyrimidine dimers in the DNA of wild carrot protoplasts. *Nature*.

[85] Eastwood A. C., McLennan A. G. (1985). Repair replication in ultraviolet light-irradiated protoplasts of *Daucus carota*. *Biochimica et Biophysica Acta: Gene Structure and Expression*.

[86] Hays J. B. (2002). *Arabidopsis thaliana*, a versatile model system for study of eukaryotic genome-maintenance functions. *DNA Repair*.

[87] Cannon G. C., Hedrick L. A., Heinhorst S. (1995). Repair mechanisms of UV-induced DNA damage in soybean chloroplasts. *Plant Molecular Biology*.

[88] Taylor R. M., Nikaido O., Jordan B. R., Rosamond J., Bray C. M., Tobin A. K. (1996). Ultraviolet-B-induced DNA lesions and their removal in wheat (*Triticum aestivum* L) leaves. *Plant, Cell and Environment*.

[89] Batty D. P., Wood R. D. (2000). Damage recognition in nucleotide excision repair of DNA. *Gene*.

[90] Maillard O., Camenisch U., Blagoev K. B., Naegeli H. (2008). Versatile protection from mutagenic DNA lesions conferred by bipartite recognition in nucleotide excision repair. *Mutation Research*.

[91] Boubriak I., Kargiolaki H., Lyne L., Osborne D. J. (1997). The requirement for DNA repair in desiccation tolerance of germinating embryos. *Seed Science Research*.

[92] The Arabidopsis Genome Initiative (TAGI) (2000). Analysis of the genome sequence of the flowering plant *Arabidopsis thaliana*. *Nature*.

[93] Caldecott K. W. (2001). Mammalian DNA single-strand break repair: an X-ra(y)ted affair. *BioEssays*.

[94] Fromme J. C., Banerjee A., Verdine G. L. (2004). DNA glycosylase recognition and catalysis. *Current Opinion in Structural Biology*.

[95] Harrington J. J., Lieber M. R. (1994). Functional domains within FEN-1 and RAD2 define a family of structure-specific endonucleases: implications for nucleotide excision repair. *Genes & Development*.

[96] Wu X., Li J., Li X., Hsieh C.-L., Burgers P. M. J., Lieber M. R. (1996). Processing of branched DNA intermediates by a complex of human FEN-1 and PCNA. *Nucleic Acids Research*.

[97] Klungland A., Lindahl T. (1997). Second pathway for completion of human DNA base excision-repair: reconstitution with purified proteins and requirement for DNase IV (FEN1). *The EMBO Journal*.

[98] García-Ortiz M.-V., Ariza R. R., Roldán-Arjona T. (2001). An OGG1 orthologue encoding a functional 8-oxoguanine DNA glycosylase/lyase in *Arabidopsis thaliana*. *Plant Molecular Biology*.

[99] Kimura S., Sakaguchi K. (2006). DNA repair in plants. *Chemical Reviews*.

[100] Kunkel T. A., Bebenek K. (2000). DNA replication fidelity. *Annual Review of Biochemistry*.

[101] Leonard J. M., Bollmann S. R., Hays J. B. (2003). Reduction of stability of Arabidopsis genomic and transgenic DNA-repeat sequences (microsatellites) by inactivation of AtMSH2 mismatch-repair function. *Plant Physiology*.

[102] Wu S.-Y., Culligan K., Lamers M., Hays J. (2003). Dissimilar mispair-recognition spectra of *Arabidopsis* DNA-mismatch-repair proteins MSH2·MSH6 (MutS*α*) and MSH2·MSH7 (MutS*γ*). *Nucleic Acids Research*.

[103] Puchta H., Dujon B., Hohn B. (1996). Two different but related mechanisms are used in plants for the repair of genomic double-strand breaks by homologous recombination. *Proceedings of the National Academy of Sciences of the United States of America*.

[104] Puchta H. (2005). The repair of double-strand breaks in plants: mechanisms and consequences for genome evolution. *The Journal of Experimental Botany*.

[105] West C. E., Waterworth W. M., Sunderland P. A., Bray C. M. (2004). Arabidopsis DNA double-strand break repair pathways. *Biochemical Society Transactions*.

[106] Jiang C.-Z., Yee J., Mitchell D. L., Britt A. B. (1997). Photorepair mutants of arabidopsis. *Proceedings of the National Academy of Sciences of the United States of America*.

[107] Liu Z., Hall J. D., Mount D. W. (2001). *Arabidopsis UVH3* gene is a homolog of the *Saccharomyces cerevisiae RAD2* and human *XPG* DNA repair genes. *Plant Journal*.

[108] Bleuyard J.-Y., Gallego M. E., Savigny F., White C. I. (2005). Differing requirements for the *Arabidopsis Rad51* paralogs in meiosis and DNA repair. *The Plant Journal*.

[109] Morgante P. G., Berra C. M., Nakabashi M., Costa R. M. A., Menck C. F. M., van Sluys M.-A. (2005). Functional *XPB/RAD25* redundancy in Arabidopsis genome: characterization of *AtXPB2* and expression analysis. *Gene*.

[110] Liang L., Flury S., Kalck V., Hohn B., Molinier J. (2006). CENTRIN2 interacts with the arabidopsis homolog of the human XPC protein (AtRAD4) and contributes to efficient synthesis-dependent repair of bulky DNA lesions. *Plant Molecular Biology*.

[111] Vonarx E. J., Tabone E. K., Osmond M. J., Anderson H. J., Kunz B. A. (2006). *Arabidopsis* homologue of human transcription factor IIH/nucleotide excision repair factor p44 can function in transcription and DNA repair and interacts with AtXPD. *The Plant Journal*.

[112] Aylon Y., Kupiec M. (2004). DSB repair: the yeast paradigm. *DNA Repair*.

[113] Krogh B. O., Symington L. S. (2004). Recombination proteins in yeast. *Annual Review of Genetics*.

[114] Schuermann D., Molinier J., Fritsch O., Hohn B. (2005). The dual nature of homologous recombination in plants. *Trends in Genetics*.

[115] Gisler B., Salomon S., Puchta H. (2002). The role of double-strand break-induced allelic homologous recombination in somatic plant cells. *Plant Journal*.

[116] Caryl A. P., Jones G. H., Franklin F. C. H. (2003). Dissecting plant meiosis using *Arabidopsis thaliana* mutants. *Journal of Experimental Botany*.

[117] Symington L. S. (2002). Role of RAD52 epistasis group genes in homologous recombination and double-strand break repair. *Microbiology and Molecular Biology Reviews*.

[118] Puizina J., Siroky J., Mokros P., Schweizer D., Riha K. (2004). *Mre11* deficiency in *Arabidopsis* is associated with chromosomal instability in somatic cells and *Spo11*-dependent genome fragmentation during meiosis. *Plant Cell*.

[119] Osakabe K., Abe K., Yoshioka T. (2006). Isolation and characterization of the *RAD54* gene from *Arabidopsis thaliana*. *The Plant Journal*.

[120] Kovalchuk I., Abramov V., Pogribny I., Kovalchuk O. (2004). Molecular aspects of plant adaptation to life in the Chernobyl zone. *Plant Physiology*.

[121] Akutsu N., Iijima K., Hinata T., Tauchi H. (2007). Characterization of the plant homolog of Nijmegen breakage syndrome 1: involvement in DNA repair and recombination. *Biochemical and Biophysical Research Communications*.

[122] Waterworth W. M., Altun C., Armstrong S. J. (2007). NBS1 is involved in DNA repair and plays a synergistic role with ATM in mediating meiotic homologous recombination in plants. *Plant Journal*.

[123] Gorbunova V., Levy A. A. (1997). Non-homologous DNA end joining in plant cells is associated with deletions and filler DNA insertions. *Nucleic Acids Research*.

[124] Goettel W., Messing J. (2009). Change of gene structure and function by non-homologous end-joining, homologous recombination, and transposition of DNA. *PLoS Genetics*.

[125] Kirik A., Salomon S., Puchta H. (2000). Species-specific double-strand break repair and genome evolution in plants. *The EMBO Journal*.

[126] Weterings E., Chen D. J., William J. L., Lane M. D. (2013). Non-homologous end joining in eukaryotes. *Encyclopedia of Biological Chemistry*.

